# The Predictive Value of Time-Varying Noninvasive Scores on Long-Term Prognosis of NAFLD in South Korea

**DOI:** 10.1155/2024/5667986

**Published:** 2024-09-16

**Authors:** Sung Won Chung, Min Kyung Park, Xiao Zhang, Tongtong Wang, Thomas Jemielita, Gail Fernandes, Samuel S. Engel, Heejoon Jang, Yun Bin Lee, Eun Ju Cho, Jeong-Hoon Lee, Su Jong Yu, Jung-Hwan Yoon, Yoon Jun Kim

**Affiliations:** ^1^ Department of Internal Medicine and Liver Research Institute Seoul National University College of Medicine, Seoul, Republic of Korea; ^2^ Department of Gastroenterology Liver Center Asan Medical Center University of Ulsan College of Medicine, Seoul, Republic of Korea; ^3^ Department of Internal Medicine Seoul National University Bundang Hospital Seoul National University College of Medicine, Seoul, Republic of Korea; ^4^ Merck & Co., Inc., Rahway, NJ, USA; ^5^ Department of Internal Medicine Seoul Metropolitan Government Seoul National University Boramae Medical Center, Seoul, Republic of Korea

## Abstract

**Background:**

This study aimed to examine whether repeated measurements on noninvasive fibrosis scores during follow-up improve long-term nonalcoholic fatty liver disease (NAFLD) outcome prediction.

**Methods:**

A cohort study of 2,280 NAFLD patients diagnosed at the Seoul National University Hospital from 2001 to 2015 was conducted. Multivariable Cox regression models with baseline and designated time-point measurements of the fibrosis-4 index (FIB-4) and NAFLD fibrosis score (NFS) were used to assess the association between these scores and overall mortality, liver-related outcomes, and cardiovascular events.

**Results:**

Higher baseline NFS (high versus low probability for advanced fibrosis groups) was associated with higher risk of mortality (adjusted hazard ratio (aHR), (95% confidence interval (CI)), 2.80, [1.39–5.63]) and liver-related outcomes (3.70, [1.27–10.78]). Similar findings were observed for the association of baseline FIB-4 with mortality (2.49, [1.46–4.24]) and liver-related outcomes (11.50, [6.17–21.44]). In models considering designated time-point measurements of the scores, stronger associations were noted. For NFS, a higher time-point measurement was associated with a significantly higher risk of mortality (3.01, [1.65–5.49]) and liver-related outcomes (6.69, [2.62–17.06]). For FIB-4, higher time-point measurements were associated with significantly higher mortality (3.01, [1.88–4.82]) and liver-related outcomes (13.26, [6.89–25.53]). An annual increase in FIB-4 (2.70, [1.79–4.05]) or NFS (4.68, [1.52–14.44]) was associated with an increased risk of liver-related outcomes. No association between NFS/FIB-4 and risk of cardiovascular events was observed in both models.

**Conclusions:**

Higher aHRs describing the associations of FIB-4/NFS with overall mortality and liver-related outcomes were observed in the models that included designated time-point measurements of the scores. In addition to the baseline measurement, a routine monitoring on these scores may be important in predicting prognosis of NAFLD patients.

## 1. Introduction

Nonalcoholic fatty liver disease (NAFLD) is the most common liver diseases worldwide [1]. The overall global prevalence of NAFLD is 30.1% based on meta-analysis of 92 population studies from 1990 to 2019 [2, 3].

Although highly prevalent, most patients with NAFLD do not develop clinically relevant liver disease [4, 5]. A subset of patients with NAFLD develops advanced fibrosis, with risk of progression to cirrhosis and hepatocellular carcinoma (HCC), which can lead to liver-related morbidity and mortality [6–8]. The most important prognostic factor for liver-related diseases and mortality in NAFLD is the fibrosis stage [9]. Previous longitudinal studies have shown that advanced fibrosis (i.e., stage 3-4 by liver biopsy) was the major predictor of clinically significant liver-related outcomes [10]. Individuals without advanced fibrosis had a lower risk of progression to cirrhosis within a 10–15-year time frame [11], whereas those with advanced fibrosis more frequently experienced severe liver-related endpoints and had higher overall mortality [12]. However, as an invasive procedure, liver biopsy is not routinely performed, and serial repeated follow-up with liver biopsies is less common. Because of that, various scoring systems have been developed to identify patient with a high probability of advanced fibrosis based on data routinely collected in clinical setting, and fibrosis-4 index (FIB-4), aspartate aminotransferase to platelet ratio index (APRI), and NAFLD Fibrosis score (NFS) are among those widely used [13].

Population-based observational studies have found that higher NFS, FIB-4, and APRI scores were associated with increased risk of liver disease and overall mortality among patients with NAFLD [14–17]. Furthermore, a retrospective cohort study found that an increase in the FIB-4 over time was associated with higher risk of severe liver disease while a decrease in the FIB-4 was associated with reduced risk [18]. However, it remains unclear if the repeated measurements on these scores can improve the prediction of long-term clinical outcomes.

In this study, we evaluated prognosis in 2,280 NAFLD patients with a median follow-up of over 10 years and assessed whether repeated measurements on NFS, FIB-4, and APRI could enhance the prediction of overall mortality, liver-related outcomes, and cardiovascular events.

## 2. Materials and Methods

### 2.1. Patients

**Figure 1:**
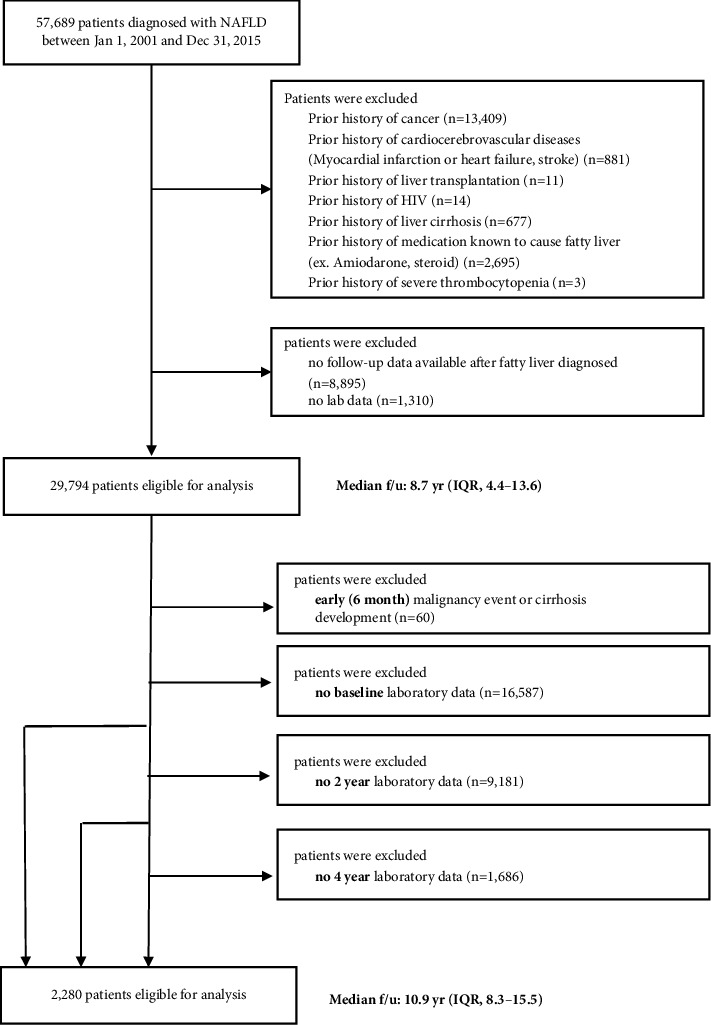
Flowchart of the patient selection process. HIV, human immunodeficiency virus; NAFLD, nonalcoholic fatty liver disease.

A retrospective cohort study of adult NAFLD patients diagnosed from 2001 to 2015 at the Seoul National University Hospital (SNUH) was conducted. NAFLD was initially defined as patients with fatty liver and without significant alcohol intake (≥210 g per week for men and ≥140 g per week for women). Fatty liver was identified if liver echogenicity surpassed that of the renal cortex and spleen, accompanied with ultrasound wave attenuation, loss of diaphragm definition, and poor delineation of intrahepatic architecture [19]. In the absence of ultrasound data, precontrast computed tomography pictures were employed. If the liver's attenuation was at least 10 Hounsfield units smaller than that of the spleen or 40 Hounsfield units, fatty liver was found [20]. Patients with chronic hepatitis B and chronic hepatitis C were not included. NAFLD patients who met any of the following criteria were also excluded: prior history of liver cirrhosis, prior history of medication known to cause fatty liver, prior history of liver transplantation, prior history of cancer, prior history of cardiocerebrovascular disease, prior history of human immunodeficiency virus infection, prior history of severe thrombocytopenia, early malignancy event or cirrhosis development, and patients with incomplete laboratory data (Figure 1). Baseline characteristics of included and excluded NAFLD patients are summarized in Supplementary Table 1. Recently, the focus has shifted from defining NAFLD to emphasizing metabolic-associated aspects, leading to the frequent use of the term metabolic dysfunction-associated steatotic liver disease (MASLD). This term avoids stigmatizing language such as “nonalcoholic” and “fatty liver” [21]. Consequently, in this study, we have identified a subpopulation of NAFLD patients who meet the criteria for MASLD. The Institutional Review Board of Seoul National University Hospital approved the study protocol, and it was conducted in accordance with the Declaration of Helsinki (approval no.: H-2104-044-1210). Because of the retrospective nature of this study, informed consent was waived.

### 2.2. Outcomes and Variables

The primary outcomes were overall mortality, liver-related outcomes, and cardiovascular events. Overall mortality was determined based on database from the Ministry of the Interior and Safety of Korea. Liver-related outcomes included cirrhosis, decompensated cirrhosis, liver transplantation, and HCC. Any history of admission or visiting outpatient department more than once with the diagnosis of cirrhosis with the following was defined as cirrhosis: (1) histological findings, (2) ultrasonographic findings (nodules in hepatic parenchyma, splenomegaly (>12 cm), or enlarged portal vein (>16 mm)), or (3) endoscopic findings compatible to cirrhosis. Decompensated cirrhosis was defined as new onset of clinically obvious ascites, overt encephalopathy, or variceal hemorrhage [22]. HCC was defined according to American association of the study of liver diseases [23] and European association of the study of liver HCC guidelines [24]. Patients newly diagnosed with acute coronary syndrome (ACS), heart failure (HF), stroke, or peripheral arterial disease (PAD) were considered cardiovascular events. All these cardiovascular events were diagnosed according to their clinical criteria, and their outcomes were initially screened through diagnosis codes (international classification of diseases (ICD)-10 codes I21, I50, I63, and I73.9) and confirmed by physicians' review of electronic medical records.

The index date was defined as the time of the first diagnosis of NAFLD during the study period. Follow-up was ended at the time of an event, emigration, death, or end of follow-up (December 31, 2021), whichever came first. Patients were also censored at the time when significant alcohol intake was documented during the follow-up period. Patients' demographic information and comorbidities were collected at the baseline. Repeated measurements of FIB-4, APRI, and NFS were collected at follow-up visit in year 2 and year 4. Calculations for FIB-4, APRI, and NFS and categorization for low, intermediate, and high probability of advanced fibrosis were described elsewhere [25–27]. Specifically, for FIB-4, two cutoff points were selected to categorize subjects into 3 groups: low (age under 60, FIB-4 <1.30; age over 60, FIB-4 <2.00), intermediate (age under 60, FIB-4: 1.30–2.67; age over 60, FIB-4: 2.00–2.67), and high (FIB-4 ≥2.67) probability of advanced fibrosis [27]. For APRI, two cutoff points were selected to categorize subjects into three groups as follows: low (APRI <0.5), intermediate (APRI: 0.5 to <1.5), and high (APRI ≥1.5) probability of advanced fibrosis [28]. For NFS, two cutoff points were selected to categorize subjects into 3 groups as follows: low (NFS < −1.455), intermediate (NFS: −1.455 to < 0.676), and high (NFS ≥0.676) probability of advanced fibrosis [26]. Annual changes in FIB-4, NFS, and APRI scores were analyzed to assess their impact on main clinical outcomes.

### 2.3. Statistical Analysis

Patients' characteristics were described using means with the standard deviations (SD), median with interquartile range (IQR), or as total numbers with percentages where applicable. The cumulative incidence of outcomes was calculated as the number of events divided by the number of person-years during the study period. For patients with missingness in laboratory data, we conducted multiple imputations for missing values under the assumption that the missing data were random using the multiple imputation with chained equation (MICE) method to analyze baseline and designated time-point measurements of NFS, FIB-4, and APRI as part of the sensitivity analysis [29].

Multivariable Cox regression models incorporating baseline and designated time-point measurements of NFS and FIB-4 were separately fit to the data to assess the association between the measurements of these scores at baseline/designated time-points and the primary outcomes. The variables included in the multivariable Cox regression models, besides FIB-4, NFS, and APRI, were age, sex, baseline body mass index (BMI) [30], baseline type II diabetes [31], baseline hyperlipidemia [32], and baseline hypertension [33], which are commonly known as prognostic risk factors of NAFLD. The variables were predetermined based on existing literature. Analyses were performed using R 4.2.0 (R Foundation for Statistical Computing, Vienna, Austria). All statistical tests were two sided, and to account for multiple comparisons (*n* = 54), Bonferroni correction was applied. Statistical significance was determined at *P* < 0.001 after correction.

## 3. Results

### 3.1. Baseline Characteristics

2,280 individuals diagnosed with NAFLD between January 2001 and December 2015 were eligible for inclusion in the study (Figure 1).  Table 1 summarizes the characteristics of patients. The median follow-up was 10.9 years (IQR: 8.3–15.5). The mean age was 55.1 years, and 56.9% were overweight or obese (BMI >25). The mean scores for the NFS and FIB-4 were −1.27 and 1.27, respectively. When MICE was performed, similar results were reproduced (Supplementary Table 2).

### 3.2. Overall Mortality

As shown in Table 2, the cumulative mortality was 0.82 cases per 100 person-years.

In the model with the baseline value of scores considered, patients with higher FIB-4 had a higher risk of death (as continuous variables, adjusted hazard ratio (aHR) = 1.23, 95% confidence interval (CI) = 1.09–1.39, and *P* < 0.001; high versus low probability of advanced fibrosis groups, aHR = 2.45, 95% CI = 1.44–4.17, and *P* < 0.001). Higher baseline NFS was associated with an increased risk of death (as continuous variables, aHR = 1.36, 95% CI = 1.13–1.64, and *P*=0.001; high versus low probability of advanced fibrosis groups, aHR = 2.96, 95% CI = 1.47–5.95, and *P*=0.002). Higher APRI also tend to have higher mortality (high versus low probability groups, aHR = 3.02, 95% CI = 1.31–6.95, and *P*=0.009) (Table 3).

In the model with designated time-point measurements of scores considered, higher aHRs were observed. Patients with higher designated time-point measurements NFS had an increased risk of death (as continuous variables, aHR = 1.43, 95% CI = 1.20–1.39, and *P* < 0.001; high versus low probability of advanced fibrosis groups, aHR = 2.97, 95% CI = 1.62–5.44, and *P* < 0.001). Higher APRI was associated with an increased risk of death (as continuous variables, aHR = 1.80, 95% CI = 1.46–2.23, and *P* < 0.001; intermediate versus low probability of advanced fibrosis groups, aHR = 1.94, 95% CI = 1.19–3.17, and *P* = 0.008; high versus low probability of advanced fibrosis groups, aHR = 8.55, 95% CI = 3.41–21.44, and *P* < 0.001). Higher FIB-4 was also associated with an increased risk of death (as high versus low probability of advanced fibrosis groups, aHR = 2.88, 95% CI = 1.79–4.63, and *P* < 0.001) (Table 3). No association of NFS or FIB-4 with risk of death was observed for the comparison between intermediate and low probability of advanced of fibrosis groups, in both models. We observed similar results when missing values were imputed (Table 4).

No association of NFS or FIB-4 with risk of death was observed for the comparison between intermediate and low probability of advanced of fibrosis groups, in both models.

### 3.3. Liver-Related Outcomes

As shown in Table 2, during follow-up, 10 HCC cases and 1 liver transplantation occurred. 126 patient developed cirrhosis, with a cumulative incidence of 0.50 cases per 100 person-years.

In the model with baseline value of scores considered, patients with higher baseline NFS tend to have a higher risk of liver-related outcomes (high versus low probability of advanced fibrosis groups, aHR = 3.79, 95% CI = 1.32–10.92, and *P*=0.01). No relationship between NFS and the risk of liver-related outcomes was found when comparing the intermediate and low probability groups for advanced fibrosis, or when NFS was analyzed as a continuous variable. Similarly, for FIB-4, a higher baseline value correlated with a higher risk of liver-related outcomes (as continuous variables, aHR = 1.35, 95% CI = 1.20–1.53, and *P* < 0.001; high versus low probability of advanced fibrosis groups, aHR = 12.08, 95% CI = 6.41–22.77, and *P* < 0.001; intermediate versus low probability of advanced fibrosis groups, aHR = 1.91, 95% CI = 1.11–3.27, and *P*=0.02). No statistically significant relationship between FIB-4 and the risk of liver-related outcomes was found when comparing the intermediate and low probability groups for advanced fibrosis. Higher baseline APRI values were also associated with an increased risk of liver-related outcomes (as continuous variables, aHR = 1.35, 95% CI = 1.13–1.61, and *P* < 0.001; high versus low probability of advanced fibrosis groups, aHR = 10.98, 95% CI = 4.99–24.15, and *P* < 0.001; intermediate versus low probability of advanced fibrosis groups, aHR = 3.81, 95% CI = 2.34–6.18, and *P* < 0.001) (Table 3).

In the model with designated time-point measurement values of scores considered, stronger associations than those in the models with only baseline value considered were observed. Patients with higher NFS at designated time-points had an increased risk of liver-related outcomes (high versus low probability of advanced fibrosis groups, aHR = 6.55, 95% CI = 2.46–17.42, and *P* < 0.001). No association between NFS and liver-related outcomes was observed for comparisons between intermediate and low risk of advanced fibrosis groups or when NFS was considered as a continuous variable. For FIB-4, higher designated time-point measurement values was associated with an increased risk of liver-related outcomes (as continuous variables, aHR = 1.38, 95% CI = 1.26–1.52, and *P* < 0.001; high versus low probability of advanced fibrosis groups, aHR = 13.65, 95% CI = 6.84–27.27, and *P* < 0.001; intermediate versus low probability of advanced fibrosis groups, aHR = 1.94, 95% CI = 1.14–3.33, and *P*=0.01). For APRI, higher designated time-point measurement values were associated with an increased risk of liver-related outcomes (as continuous variables, aHR = 1.78, 95% CI = 1.48–2.14, and *P* < 0.001; high versus low probability of advanced fibrosis groups, aHR = 11.73, 95% CI = 5.02–27.45, and *P* < 0.001; intermediate versus low probability of advanced fibrosis groups, aHR = 4.31, 95% CI = 2.69–6.90, and *P* < 0.001) (Table 3). We observed similar results when missing values were imputed (Table 4).

### 3.4. Cardiovascular Events

As shown in Table 2, cumulative incidence of ACS, HF, stroke, and PAD were 0.16, 0.24, 0.73, and 0.26 per 100 person-years, respectively. There does not appear to be an association between NFS/FIB-4 and the risk of cardiovascular events, for the comparison between high versus low or intermediate versus low probability of advanced fibrosis groups, in both models (Table 3).

### 3.5. Annual Changes in FIB-4, APRI, and NFS Scores and Their Impact on Outcomes

Over a maximum follow-up of four years, changes in FIB-4 (0.02 [−0.02–0.07]/year), NFS (−0.005 [−0.18–0.12]/year), and APRI (−0.008 [−0.07–0.05]/year) were not substantial. However, the analysis of changes in FIB-4, NFS, and APRI scores demonstrated significant associations with various outcomes (Table 5). Specifically, an increase in the FIB-4 score (adjusted hazard ratio (aHR) = 2.49; 95% confidence interval (CI) = 1.61–3.86; and *P* < 0.001) and APRI score (aHR = 2.01; 95% CI = 1.52–2.65; and *P* < 0.001) were associated with a significantly higher risk of overall mortality. In contrast, changes in NFS scores were not significantly associated with mortality (aHR = 1.88; 95% CI = 0.90–3.90; and *P* = 0.09). However, increases in both FIB-4 (aHR = 2.70; 95% CI = 1.79–4.05; and *P* < 0.001) and NFS (aHR = 4.68; 95% CI = 1.52–14.44; and *P* = 0.007) scores were significantly associated with higher risks of liver-related outcomes. Increases in APRI scores were not significantly associated with liver-related outcomes. Neither scoring system showed a significant association with cardiovascular outcomes, indicating that increases in FIB-4, NFS, and APRI scores are not linked to an increased risk of cardiovascular events.

### 3.6. MASLD Patients and Clinical Outcomes

The MASLD subpopulation included 2,272 individuals, representing 99.6% of the entire NAFLD patient group (Supplementary Table 1). The outcomes replicated were nearly identical to those observed in NAFLD (Supplementary Table 2).

## 4. Discussion

In this retrospective cohort study of 2,280 NAFLD patients with a median follow-up of over 10 years, we assessed the association between NFS/FIB-4/APRI and risk of death, liver-related outcomes, and cardiovascular events, with considering the longitudinal changes of these scores. We found that patients with higher designated time-point measurements of NFS/FIB-4/APRI scores had a higher risk of liver-related outcomes compared to those with lower scores. Significant associations were observed in the designated time-point measurements of NFS, FIB-4, and APRI after adjusting for multiple comparisons using the Bonferroni correction. There does not appear to be an association between NFS/FIB-4/APRI and the risk of cardiovascular events in both models. Following recent definitions, it has been demonstrated that NFS/FIB-4/APRI scores provide consistent results in patients with MASLD as well as NAFLD, suggesting their potential utility in future studies.

NFS, FIB-4, and APRI were among commonly used noninvasive tests to rule out advanced fibrosis in at-risk groups [34]. Higher baseline values were associated with a higher risk of all-cause mortality and liver-related outcomes [34, 35]. With repeated measurement available in our cohort, we were able to use advanced statistical approach to evaluate the prognostic value of the designated time-point measurements scores on long-term clinical outcomes. As compared to baseline measurement, scores measured more recently may be more relevant to the onset of events and better predict the patient's risk of future events. We observed a significant increase in risk of death and liver-related outcomes comparing patients with high probability of advanced fibrosis to those with low probability. The difference in risk of outcomes when comparing intermediate to low probability groups was not as significant, suggesting that those with higher probability of advanced fibrosis were more likely to experience outcomes. Unlike liver biopsy, the parameters used to calculate these scores are routinely collected and easily accessible in clinical setting, making regularly monitoring on these scores and risk-stratification of patients in regard to long-term prognosis possible to inform targeted interventions.

NAFLD is a multisystem disease, and its clinical burden is not limited to the liver. NAFLD is closely associated with subclinical markers of cardiovascular disease and advanced fibrosis in NAFLD patients was reported to be an independent cardiovascular risk factor [36, 37]. As easy to calculate and widely available scores to quantify the probability of advanced fibrosis, NFS, FIB-4, and APRI have the potential as a prognostic score for cardiovascular risk [38, 39]. In our study, a positive association between the designated time-point measurements of NFS, FIB-4, and APRI and cardiovascular risk was observed, but the results were not statistically significant. Previous studies have reported inconsistent findings regarding the association between these scoring systems and cardiovascular outcomes [34, 38, 39]. The discrepancy in the findings may be attributed to the difference in sample size, definitions of cardiovascular events, inclusion/exclusion criteria of the study population, statistical modelling, and management of effect modifiers in the analyses.

We estimated incidence of primary outcomes in our NAFLD cohort. In a previous study conducted in the US, the incidence of HCC in western population with no cirrhosis was 0.03 cases per 100 person-years [40]. This is in line with our study findings. Another study in Sweden [41] found that NAFLD patients had a higher risk of overall mortality and liver-related outcomes than non-NAFLD patients, but the incidence of overall mortality and liver-related outcomes were higher than our current study. This difference may be explained by underlying difference in study populations or ascertainment methods on outcomes. Of note, direct comparison might be challenging due to the variation in the severity of NAFLD and other baseline variables.

This study is the first to evaluate the predictive value of designated time-point measurements of NFS, FIB-4, and APRI on the long-term prognosis of NAFLD patients in South Korea. However, limitations of this study should be noted. First, although noninvasive fibrosis indices such as NFS, FIB-4, and APRI have been validated in a heterogeneous group of NAFLD patients and have relatively good accuracy in detecting advanced fibrosis (F3 or F4), their components include routine blood tests that can be affected by other factors other than the degree of liver fibrosis, hence decreasing their accuracy. Second, although higher risk estimates were observed in models that considered designated time-point measurements, the magnitude of difference compared to the models with baseline values was small, likely because the follow-up measurements on the scores were taken close to the baseline. We excluded patients with incomplete follow-up, potentially introducing selection bias and limiting the external validity of the study. Future longitudinal studies are warranted to validate our study findings. In addition, for patients with follow-up but incomplete laboratory data, we utilized multiple imputations to address missingness in our analysis, assuming it to be missing at random. However, it is important to acknowledge that there is a possibility that the missingness is not at random, which could introduce bias to the results. Lastly, this investigation was conducted retrospectively at a single tertiary center, and results of this study may not be generalized to patients with different characteristics.

In conclusion, this retrospective cohort study demonstrated that baseline and designated time-point measurements values of FIB-4, NFS, and APRI were significantly associated with liver-related outcomes in NAFLD or MASLD patients. The results indicate that routine monitoring of these scores in addition to the baseline measurement may better predict the prognosis of NAFLD or MASLD patients. Further studies are needed to validate the findings in diverse populations.

## Figures and Tables

**Table 1:** Baseline characteristics of the patients.

	Total (*n* = 2280)^†^
	Mean ± SD
	*N* (%)
Median (IQR) follow-up, years	10.9 (8.3–15.5)
Demographics	
Age, years	55.1 ± 10.4
Male	1432 (62.8%)
Anthropometric measurements	
BMI	
Continuous	25.7 ± 3.0
Underweight (<18.5 kg/m^2^)	6 (0.3%)
Normal (18.5–22.9 kg/m^2^)	326 (16.8%)
Overweight (23.0–24.9 kg/m^2^)	502 (25.9%)
Obese (25.0–29.9 kg/m^2^)	940 (48.5%)
Severely obese (≥30.0 kg/m^2^)	163 (8.4%)
Comorbidity	
Hypertension	698 (30.6%)
Dyslipidemia	422 (18.5%)
Diabetes mellitus	647 (28.4%)
Osteoarthritis	21 (0.9%)
Osteoporosis	29 (1.3%)
Depression	11 (0.5%)
Lab	
Hb (g/dL)	14.7 ± 1.6
Plt (×10^3^/*μ*L)	240.1 ± 58.0
Neutrophil/lymphocyte	1.8 ± 1.2
ALT (U/L)	38.5 ± 35.3
AST (U/L)	30.9 ± 25.7
ALP (IU/L)	68.9 ± 25.7
GGT (IU/L)	54.0 ± 87.2
Bilirubin (mg/dL)	1.0 ± 0.5
HDL (mg/dL)	48.9 ± 11.5
Creatinine (mg/dL)	1.02 ± 0.80
HbA1c (%)	6.4 ± 1.3
Albumin (g/dL)	4.4 ± 0.3
Scoring system	
NFS^∗∗^	
Continuous	−1.27 ± 1.03
Low	807 (42.0%)
Intermediate	1068 (55.6%)
High	46 (2.4%)
FIB-4^∗∗∗^	
Continuous	1.27 ± 0.77
Low	1817 (79.7%)
Intermediate	377 (16.5%)
High	86 (3.8%)
APRI^∗∗∗∗^	
Continuous	0.35 ± 0.36
Low	1997 (87.6%)
Intermediate	255 (11.2%)
High	28 (1.2%)

^†^Some groups may not add up to the total due to missing values. ALP, alkaline phosphatase; ALT, alanine transferase; APRI, AST-to-platelet ratio; AST, aspartate transferase; BMI, body mass index; FIB-4, fibrosis index-4; GGT, gamma-glutamyl transferase; Hb, hemoglobin; HbA1c, hemoglobin A1c; HDL, high density lipoprotein; IQR, interquartile range; NFS, nonalcoholic fatty liver disease fibrosis score; Plt, platelet; SD, standard deviation. Definitions: ^∗∗^*N*FS high: ≥0.676 intermediate: −1.455 to <0.676 low: <−1.455. ^∗∗∗^FIB-4 high: ≥2.67 intermediate: 1.3 (2.0 in age >60) to <2.67 low: <1.3 (2.0 in age >60). ^∗∗∗∗^APRI high: ≥1.5 intermediate: 0.5 to <1.5 low: <0.5.

**Table 2:** Incidence of primary outcomes during the follow-up.

	Number of events^∗^	Person-years	Cumulative incidence, per 100 person-years
Deaths	216	26452.83	0.82
Liver-related outcome			
HCC	10	26048.17	0.04
Liver transplantation	1	26061.55	0.004
Cirrhosis	126	25225.75	0.50
Decompensated cirrhosis	31	25815.56	0.12
Cardiovascular events			
ACS	42	25723.41	0.16
HF	61	25846.17	0.24
Stroke	180	24643.13	0.73
PAD	68	25845.18	0.26

^∗^One patient may have multiple events. ACS, acute coronary syndrome; HCC, hepatocellular carcinoma; HF, heart failure; IQR, interquartile range; PAD, peripheral artery disease.

**Table 3:** Multivariable Cox regression of scoring systems on risk of primary outcomes.

	Death			Liver-related outcomes			Cardiovascular events		
	aHR^∗^	95% CI	*P*	aHR^∗^	95% CI	*P*	aHR^∗^	95% CI	*P*
*NFS* ^∗∗^									
Baseline NFS (continuous)	1.36	1.13–1.64	0.001	0.97	0.75–1.24	0.78	0.92	0.79–1.08	0.30
Baseline NFS (intermediate vs. low)	1.13	0.73–1.74	0.59	0.82	0.49–1.38	0.46	0.94	0.72–1.24	0.69
Baseline NFS (high vs. low)	2.96	1.47–5.95	0.002	3.79	1.32–10.92	0.01	1.06	1.05–1.08	<0.001
Designated time-point measurements NFS (continuous)	1.43	1.20–1.70	<0.001	1.18	0.95–1.46	0.14	0.95	0.82–1.09	0.47
Designated time-point measurements NFS (intermediate vs. low)	1.22	0.78–1.91	0.37	1.15	0.69–1.93	0.59	0.81	0.60–1.10	0.18
Designated time-point measurements NFS (high vs. low)	2.97	1.62–5.44	<0.001	6.55	2.46–17.42	<0.001	1.22	0.68–2.17	0.51

*FIB-4* ^∗∗∗^									
Baseline FIB-4 (continuous)	1.23	1.09–1.39	<0.001	1.35	1.20–1.53	<0.001	1.12	0.98–1.28	0.10
Baseline FIB-4 (intermediate vs. low)	0.97	0.66–1.44	0.89	1.91	1.11–3.27	0.02	1.07	0.85–1.33	0.58
Baseline FIB-4 (high vs. low)	2.45	1.44–4.17	<0.001	12.08	6.41–22.77	<0.001	1.06	1.04–1.07	<0.001
Designated time-point measurements FIB-4 (continuous)	1.19	1.11–1.28	<0.001	1.38	1.26–1.52	<0.001	1.02	0.88–1.18	0.83
Designated time-point measurements FIB-4 (intermediate vs. low)	1.28	0.86–1.92	0.22	1.95	1.14–3.33	0.01	0.92	0.68–1.25	0.61
Designated time-point measurements FIB-4 (high vs. low)	2.88	1.79–4.63	<0.001	13.65	6.84–27.27	<0.001	1.34	0.78–2.31	0.28

*APRI* ^∗∗∗∗^									
Baseline APRI (continuous)	1.26	0.97–1.64	0.08	1.35	1.13–1.61	<0.001	1.32	1.07–1.62	0.01
Baseline APRI (intermediate vs. low)	1.22	0.76–1.96	0.42	3.81	2.34–6.18	<0.001	1.49	1.05–2.13	0.03
Baseline APRI (high vs. low)	3.02	1.31–6.95	0.009	10.98	4.99–24.15	<0.001	1.69	0.69–4.13	0.25
Designated time-point measurements APRI (continuous)	1.80	1.46–2.23	<0.001	1.78	1.48–2.14	<0.001	1.08	0.74–1.57	0.71
Designated time-point measurements APRI (intermediate vs. low)	1.94	1.19–3.17	0.008	4.31	2.69–6.90	<0.001	1.46	1.01–2.12	0.04
Designated time-point measurements APRI (high vs. low)	8.55	3.41–21.44	<0.001	11.73	5.02–27.45	<0.001	0.99	0.24–3.98	0.98

aHR, adjusted hazards ratio; APRI, aspartate aminotransferase to platelet ratio; CI, confidence interval; FIB-4, fibrosis 4 index; NFS, nonalcoholic fatty liver disease fibrosis score. ^∗^Adjusting for age, sex, baseline body mass index, baseline type II diabetes, baseline hyperlipidemia, and baseline hypertension. Definitions: ^∗∗^NFS high: >0.676 intermediate: −1.455–0.676 low: <−1.455. ^∗∗∗^FIB-4 high: ≥2.67 intermediate: 1.3 (2.0 in age >60) to <2.67 low: <1.3 (2.0 in age >60). ^∗∗∗∗^APRI high: ≥1.5 intermediate: 0.5 to <1.5 low: <0.5.

**Table 4:** Multivariable Cox regression of scoring systems on risk of primary outcomes (imputed data).

	Death			Liver-related outcomes			Cardiovascular events		
	aHR^∗^	95% CI	*P*	aHR^∗^	95% CI	*P*	aHR^∗^	95% CI	*P*
*NFS* ^∗∗^									
Baseline NFS (intermediate vs. low)	1.15	0.75–1.78	0.52	0.82	0.61–1.33	0.42	0.83	0.61–1.12	0.22
Baseline NFS (high vs. low)	3.23	1.63–6.12	<0.001	4.09	1.56–10.68	0.004	1.21	0.64–2.30	0.56
Designated time-point measurements NFS (intermediate vs. low)	1.18	0.75–1.84	0.48	1.06	0.65–1.71	0.82	0.76	0.57–1.02	0.08
Designated time-point measurements NFS (high vs. low)	2.43	1.34–4.42	0.003	5.24	2.10–13.06	<0.001	1.16	0.67–2.00	0.60

*FIB-4* ^∗∗∗^									
Baseline FIB-4 (intermediate vs. low)	1.15	0.76–1.75	0.52	1.51	0.85–2.70	0.16	1.15	0.84–1.57	0.39
Baseline FIB-4 (high vs. low)	2.53	1.49–4.30	<0.001	10.94	5.87–20.39	<0.001	1.40	0.80–2.45	0.23
Designated time-point measurements FIB-4 (intermediate vs. low)	1.35	0.90–2.01	0.14	1.56	0.89–2.72	0.12	0.96	0.71–1.32	0.82
Designated time-point measurements FIB-4 (high vs. low)	3.01	1.88–4.82	<0.001	12.29	6.42–23.56	<0.001	1.29	0.74–2.26	0.37

*APRI* ^∗∗∗∗^									
Baseline APRI (intermediate vs. low)	1.28	0.81–2.05	0.29	3.78	2.32–6.14	<0.001	1.47	1.03–2.10	0.03
Baseline APRI (high vs. low)	2.98	1.30–6.84	0.01	10.76	4.91–23.58	<0.001	1.68	0.69–4.10	0.25
Designated time-point measurements APRI (intermediate vs. low)	1.90	1.14–3.17	0.01	4.16	2.56–6.78	<0.001	1.33	0.88–2.01	0.17
Designated time-point measurements APRI (high vs. low)	8.71	3.47–21.83	<0.001	14.58	6.46–32.93	<0.001	1.22	0.30–4.92	0.78

aHR, adjusted hazards ratio; APRI, aspartate aminotransferase to platelet ratio; CI, confidence interval; FIB-4, fibrosis 4 index; NFS, nonalcoholic fatty liver disease fibrosis score. ^∗^Adjusting for age, sex, baseline body mass index, baseline type II diabetes, baseline hyperlipidemia, and baseline hypertension. Definitions: ^∗∗^NFS high: >0.676 intermediate: −1.455-0.676 low: <−1.455. ^∗∗∗^FIB-4 high: ≥2.67 intermediate: 1.3 (2.0 in age >60) to <2.67 low: <1.3 (2.0 in age >60). ^∗∗∗∗^APRI high: ≥1.5 intermediate: 0.5 to <1.5 low: <0.5.

**Table 5:** Multivariable Cox regression of scoring systems on risk of primary outcomes according to annual NFS/FIB-4/APRI changes during the follow-up.

	Death			Liver-related outcomes			Cardiovascular events		
	aHR^∗^	95% CI	*P*	aHR^∗^	95% CI	*P*	aHR^∗^	95% CI	*P*
Delta NFS, per year	1.88	0.90–3.90	0.09	4.68	1.52–14.44	0.007	0.98	0.54–1.78	0.95
Delta FIB-4, per year	2.49	1.61–3.86	<0.001	2.70	1.79–4.05	<0.001	0.85	0.46–1.59	0.62
Delta APRI, per year	2.01	1.52–2.65	<0.001	1.38	0.94–2.03	0.10	0.56	0.65–1.14	0.29

aHR, adjusted hazards ratio; APRI, aspartate aminotransferase to platelet ratio; CI, confidence interval; FIB-4, fibrosis 4 index; NFS, nonalcoholic fatty liver disease fibrosis score. ^∗^Adjusting for age, sex, baseline body mass index, baseline type II diabetes, baseline hyperlipidemia, and baseline hypertension.

## Data Availability

The datasets used and/or analyzed during the current study are available from the corresponding author upon reasonable request.

## Supplementary Materials

Supplementary Materials Supplementary Table 1: baseline characteristics of excluded and included NAFLD patients. Supplementary Table 2: baseline characteristics of excluded and included NAFLD patients. Supplementary Table 3: baseline characteristics of the MASLD patients. Supplementary Table 4: multivariable Cox regression of scoring systems on risk of primary outcomes in MASLD patients.
