# Supplementary material for: The Predictive Value of Time-Varying Noninvasive Scores on Long-Term Prognosis of NAFLD in South Korea
Source: Can J Gastroenterol Hepatol. 2024 Sep 16;2024:5667986. doi: 10.1155/2024/5667986 (PMC11419836; doi:10.1155/2024/5667986)
Supplement: Supplementary Materials — Supplementary Table 1: baseline characteristics of excluded and included NAFLD patients. Supplementary Table 2: baseline characteristics of excluded and included NAFLD patients. Supplementary Table 3: baseline characteristics of the MASLD patients. Supplementary Table 4: multivariable Cox regression of scoring systems on risk of primary outcomes in MASLD patients. [file 5667986.f1.docx]

***Supplementary Material***

**The predictive value of time-varying non-invasive scores on long-term prognosis of NAFLD in South Korea**

Sung Won Chung, et al.

**Supplementary Table 1. Baseline characteristics of excluded and included NAFLD patients**

|  | Excluded patients | Included patients | P |
| --- | --- | --- | --- |
|  | (N=37,719) | (N=2280) |  |
| Age group |  |  | <0.001 |
| 19 | 141 (0.4%) | 0 (0.0%) |  |
| 20-24 | 685 (1.8%) | 6 (0.3%) |  |
| 25-29 | 1129 (3.0%) | 22 (1.0%) |  |
| 30-34 | 2028 (5.4%) | 51 (2.2%) |  |
| 35-39 | 2835 (7.5%) | 84 (3.7%) |  |
| 40-44 | 3461 (9.2%) | 181 (7.9%) |  |
| 45-49 | 4552 (12.1%) | 355 (15.6%) |  |
| 50-54 | 5702 (15.1%) | 450 (19.7%) |  |
| 55-59 | 6030 (16.0%) | 409 (17.9%) |  |
| 60-64 | 5166 (13.7%) | 325 (14.3%) |  |
| 65-69 | 3425 (9.1%) | 231 (10.1%) |  |
| 70-74 | 1724 (4.6%) | 122 (5.4%) |  |
| 75-79 | 606 (1.6%) | 26 (1.1%) |  |
| 80-84 | 193 (0.5%) | 15 (0.7%) |  |
| 85-89 | 25 (0.06%) | 2 (0.09%) |  |
| 90-94 | 7 (0.02%) | 1 (0.04%) |  |
| Sex |  |  | <0.001 |
| Female | 16,539 (43.9%) | 848 (37.2%) |  |
| Male | 21,180 (56.1%) | 1432 (62.8%) |  |
| Index year |  |  | <0.001 |
| 2001-2005 | 17,168 (45.5%) | 799 (35.0%) |  |
| 2006-2010 | 11,905 (31.6%) | 938 (41.1%) |  |
| 2011-2015 | 8646 (23.0%) | 543 (23.8%) |  |

Abbreviation NAFLD, non-alcoholic fatty liver disease.

**Supplementary Table 2. Baseline characteristics (imputed)**

|  | Total (n=2280)* |
| --- | --- |
| Age, years | 55.1 ± 10.4 |
| Male | 1432 (62.8%) |
| BMI |  |
| Continuous | 25.7 ± 3.0 |
| Underweight (<18.5 kg/m^2^) | 7 (0.3%) |
| Normal (18.5–22.9 kg/m^2^) | 398 (17.5%) |
| Overweight (23.0–24.9 kg/m^2^) | 586 (25.7%) |
| Obese (25.0–29.9 kg/m^2^) | 1100 (48.3%) |
| Severely obese (≥30.0 kg/m^2^) | 189 (8.3%) |
| NFS* |  |
| Continuous | -1.23 ± 1.05 |
| Low | 916 (40.2%) |
| Intermediate | 1296 (56.8%) |
| High | 68 (3.0%) |
| FIB-4** |  |
| Continuous | 1.27 ± 0.77 |
| Low | 1791 (78.6%) |
| Intermediate | 403 (17.7%) |
| High | 86 (3.8%) |
| APRI*** |  |
| Continuous | 0.35 ± 0.36 |
| Low | 1993 (87.4%) |
| Intermediate | 258 (11.3%) |
| High | 29 (1.3%) |

* Missing values were imputed using multiple imputation of chained equations.

Abbreviations APRI, AST to platelet ratio; BMI, body mass index; FIB-4, fibrosis index-4; NFS, non-alcoholic fatty liver disease fibrosis score.

Definitions:

** NFS high: ≥0.676 intermediate: -1.455 to <0.676 low: <-1.455

*** FIB-4 high: ≥2.67 intermediate: 1.3 (2.0 in age >60) to <2.67 low: <1.3 (2.0 in age >60)

**** APRI high: ≥1.5 intermediate: 0.5 to <1.5 low: <0.5

**Supplementary Table 3. Baseline characteristics of the MASLD patients**

|  | **Total (n=2272)*** |  |  |
| --- | --- | --- | --- |
|  | **Mean ± SD** |  |  |
|  | **N (%)** |  |  |
| **Median (IQR) follow-up, years** | 10.9 (8.3–15.5) |  |  |
| **Demographics** |  |  |  |
| **Age, years** | 55.1 ± 10.4 |  |  |
| **Male** | 1428 (62.9%) |  |  |
| **Anthropometric measurements** |  |  |  |
| **BMI** |  |  |  |
| **Continuous** | 25.7 ± 3.0 |  |  |
| **Underweight (<18.5 kg/m^2^)** | 5 (0.3%) |  |  |
| **Normal (18.5–22.9 kg/m^2^)** | 319 (16.5%) |  |  |
| **Overweight (23.0–24.9 kg/m^2^)** | 502 (26.0%) |  |  |
| **Obese (25.0–29.9 kg/m^2^)** | 940 (48.7%) |  |  |
| **Severe Obese (≥30.0 kg/m^2^)** | 163 (8.4%) |  |  |
| **Comorbidity** |  |  |  |
| **Hypertension** | 598 (31.7%) |  |  |
| **Dyslipidemia** | 369 (19.6%) |  |  |
| **Diabetes mellitus** | 515 (27.3%) |  |  |
| **Osteoarthritis** | 21 (0.9%) |  |  |
| **Osteoporosis** | 29 (1.3%) |  |  |
| **Depression** | 11 (0.5%) |  |  |
| **Scoring system** |  |  |  |
| **NFS**** |  |  |  |
| **Continuous** | -1.27 ± 1.03 |  |  |
| **Low** | 803 (42.0%) |  |  |
| **Intermediate** | 1066 (55.7%) |  |  |
| **High** | 45 (2.4%) |  |  |
| **FIB-4***** |  |  |  |
| **Continuous** | 1.26 ± 0.74 |  |  |
| **Low** | 1813 (79.8%) |  |  |
| **Intermediate** | 376 (16.5%) |  |  |
| **High** | 83 (3.7%) |  |  |
| **APRI****** |  |  |  |
| **Continuous** | 0.27 ± 0.30 |  |  |
| **Low** | 1993 (87.7%) |  |  |
| **Intermediate** | 252 (11.1%) |  |  |
| **High** | 27 (1.2%) |  |  |

* Some groups may not add up to total because of missing values.

Abbreviations ALP, alkaline phosphatase; ALT, alanine transferase; APRI, AST to platelet ratio; AST, aspartate transferase; BMI, body mass index; FIB-4, fibrosis index-4; GGT, gamma-glutamyl transferase; Hb, hemoglobin; HbA1c, hemoglobin A1c; HDL, high density lipoprotein; IQR, interquartile range; MASLD, metabolic dysfunction-associated steatotic liver disease; NFS, non-alcoholic fatty liver disease fibrosis score; Plt, platelet; SD, standard deviation.

Definitions:

** NFS high: ≥0.676 intermediate: -1.455 to <0.676 low: <-1.455

*** FIB-4 high: ≥2.67 intermediate: 1.3 (2.0 in age >60) to <2.67 low: <1.3 (2.0 in age >60)

**** APRI high: ≥1.5 intermediate: 0.5 to <1.5 low: <0.5

**Supplementary Table 4. Multivariable Cox regression of scoring systems on risk of primary outcomes in MASLD patients**

|  | **Death** | | |  | **Liver-related outcomes** | | |  | **Cardiovascular events** | | |  |
| --- | --- | --- | --- | --- | --- | --- | --- | --- | --- | --- | --- | --- |
|  | **aHR*** | **95% CI** | **P** |  | **aHR*** | **95% CI** | **P** |  | **aHR*** | **95% CI** | **P** |  |
| **NFS **** |  |  |  |  |  |  |  |  |  |  |  |  |
| Baseline NFS (continuous) | 1.32 | 1.10–1.60 | 0.004 |  | 0.94 | 0.73–1.22 | 0.65 |  | 0.92 | 0.79–1.08 | 0.30 |  |
| Baseline NFS (intermediate vs low) | 1.06 | 0.68–1.64 | 0.80 |  | 0.80 | 0.48–1.35 | 0.41 |  | 0.85 | 0.63–1.16 | 0.30 |  |
| Baseline NFS (high vs low) | 2.66 | 1.32–5.36 | 0.006 |  | 3.67 | 1.27–10.62 | 0.02 |  | 1.26 | 0.66–2.39 | 0.48 |  |
| Designated time-point measurements NFS (continuous) | 1.45 | 1.21–1.72 | <0.001 |  | 1.17 | 0.94–1.46 | 0.15 |  | 0.92 | 0.80–1.06 | 0.27 |  |
| Designated time-point measurements NFS (intermediate vs low) | 1.25 | 0.80–1.97 | 0.33 |  | 1.13 | 0.67–1.89 | 0.65 |  | 0.76 | 0.56–1.03 | 0.08 |  |
| Designated time-point measurements NFS (high vs low) | 3.11 | 1.69–5.71 | <0.001 |  | 6.52 | 2.56–16.58 | <0.001 |  | 1.11 | 0.62–1.98 | 0.73 |  |
|  |  |  |  |  |  |  |  |  |  |  |  |  |
| **FIB-4 ***** |  |  |  |  |  |  |  |  |  |  |  |  |
| Baseline FIB-4 (continuous) | 1.22 | 1.08–1.38 | 0.001 |  | 1.35 | 1.20–1.52 | <0.001 |  | 1.12 | 0.98–1.28 | 0.10 |  |
| Baseline FIB-4 (intermediate vs low) | 1.08 | 0.75–1.55 | 0.68 |  | 1.82 | 1.06–3.14 | 0.03 |  | 0.76 | 0.56–1.02 | 0.07 |  |
| Baseline FIB-4 (high vs low) | 2.57 | 1.47–4.50 | <0.001 |  | 11.66 | 6.18–22.00 | <0.001 |  | 1.16 | 0.65–2.06 | 0.62 |  |
| Designated time-point measurements FIB-4 (continuous) | 1.19 | 1.11–1.28 | <0.001 |  | 1.37 | 1.25–1.49 | <0.001 |  | 1.02 | 0.88–1.18 | 0.81 |  |
| Designated time-point measurements FIB-4 (intermediate vs low) | 1.13 | 0.76–1.66 | 0.55 |  | 2.42 | 1.43–4.09 | <0.001 |  | 0.88 | 0.66–1.18 | 0.39 |  |
| Designated time-point measurements FIB-4 (high vs low) | 3.00 | 1.76–5.10 | <0.001 |  | 15.07 | 7.73–29.41 | <0.001 |  | 1.23 | 0.70–2.18 | 0.47 |  |
|  |  |  |  |  |  |  |  |  |  |  |  |  |
| **APRI ****** | |  |  |  |  |  |  |  |  |  |  |  |
| Baseline APRI (continuous) | | 1.25 | 0.96–1.63 | 0.09 |  | 1.35 | 1.13–1.61 | <0.001 |  | 1.32 | 1.07–1.62 | 0.01 |
| Baseline APRI (intermediate vs low) | | 1.22 | 0.75–1.96 | 0.42 |  | 3.78 | 2.32–6.15 | <0.001 |  | 1.48 | 1.04–2.11 | 0.03 |
| Baseline APRI (high vs low) | | 2.86 | 1.24–6.56 | 0.01 |  | 10.59 | 4.84–23.18 | <0.001 |  | 1.68 | 0.69–4.10 | 0.25 |
| Designated time-point measurements APRI (continuous) | | 1.79 | 1.45–2.20 | <0.001 |  | 1.54 | 1.32–1.80 | <0.001 |  | 1.07 | 0.83–1.57 | 0.74 |
| Designated time-point measurements APRI (intermediate vs low) | | 1.95 | 1.20–3.18 | 0.007 |  | 4.33 | 2.70–6.96 | <0.001 |  | 1.44 | 0.99–2.09 | 0.051 |
| Designated time-point measurements APRI (high vs low) | | 8.01 | 3.20–20.06 | <0.001 |  | 12.51 | 5.52–28.35 | <0.001 |  | 0.98 | 0.24–3.97 | 0.98 |

Abbreviations: aHR, adjusted hazards ratio; APRI, aspartate to platelet ratio; CI-confidence interval; FIB-4, fibrosis 4 index; MASLD, metabolic dysfunction-associated steatotic liver disease; NFS, Non-Alcoholic Fatty Liver Disease Fibrosis Score.

* Adjusting for age, sex, baseline body mass index, baseline type II diabetes, baseline hyperlipidemia, baseline hypertension.

Definitions:

** NFS high: >0.676 intermediate: -1.455-0.676 low: <-1.455. *** FIB-4 high: ≥2.67 intermediate: 1.3 (2.0 in age >60) to <2.67 low: <1.3 (2.0 in age >60). **** APRI high: ≥1.5 intermediate: 0.5 to <1.5 low: <0.5
